# Indicators to compare and assess the institutional strength of voluntary sustainability standards in the global coffee industry

**DOI:** 10.1016/j.dib.2018.05.048

**Published:** 2018-05-16

**Authors:** Thomas Dietz, Jennie Auffenberg, Andrea Estrella Chong, Janina Grabs, Bernard Kilian

**Affiliations:** a Institute of Political Science, Westfälische Wilhelms-Universität Münster, Scharnhorststr. 100, D-48151 Münster, Germany; b University of Bremen, Bremen International Graduate School of Social Sciences, Mary-Somerville-Str. 9, D-28359 Bremen, Germany; c INCAE Business School, P.O. Box: 960-4050, La Garita, Alajuela, Costa Rica

## Abstract

The data presented in this article are related to the research article entitled “The Voluntary Coffee Standard Index (VOCSI). Developing a composite Index to Assess and Compare the Institutional Strength of Mainstream Voluntary Sustainability Standards in the Global Coffee Industry” (Dietz et al., 2018) in press) [1]. The VOCSI presents the most detailed comparison all major voluntary sustainability standards (VSS) that currently exist in global coffee production. This Data in Brief contains the database that we have generated to set up the VOCSI. We publish this dataset in order to facilitate further critical or extended analyzes.

**Specifications Table**

**Table:** 

Subject area	*Politics, Economics, Law*
More specific subject area	*Sustainability Studies, Voluntary Sustainability Standards*
Type of data	*Table (word document)*
How data was acquired	*Document analysis, Survey*
Data format	*Raw, partly analyzed*
Experimental factors	
Experimental features	
Data source location	
Data accessibility	*Data is with this article*

**Value of the data**

• Our database currently presents the most detailed comparison of all major VSS that exist in global coffee production.
• It will assist further research on the effectiveness of VSS.
• Especially, it may serve researchers to better explain impacts related to VSS and to better understand how impact and institutional characteristics are correlated.
• The database may also assist consumers and further stakeholders in business, politics and civil society to differentiate between VSS with stronger and weaker institutional designs.
• Standard setters may use the database to benchmark and improve their institutional designs.

## 1 Data

The following table (Table 1) contains a comprehensive list of regulatory topics related to sustainability transformations in the global coffee sector. The numbers show how strictly each VSS addresses each of these regulatory topics (scale: 0–3).

## 2 Experimental design, materials and methods

The creation of this dataset is based on an extensive document analysis of all standard catalogues and attached enforcement documents that the different standard setters have set up (Dietz et al. [1]). For a list of these documents, see Appendix A.

## Funding sources

This work was supported by the Land Nordrhein-Westfalen, Ministerium für Innovation, Wissenschaft und Forschung (005-1503-0008) through its financial support of the research group TRANS SUSTAIN.

## Figures and Tables

**Table:** 

**4C**	2014, 4C Code of Conduct: Approved by the 4C Council in December 9^th^, 2014, Version 2.0
**Bird friendly**	2002, Norms for Production, Processing and Marketing of “Bird Friendly^®^” Coffee – Certified Organic Shade Grown Coffee
**EU-Organic**	2007, Council Regulation (EC) No 834/2007 of 28 June 2007
	2008, Commission Regulation (EC) No 889/2008 of 5 September 2008
**Fairtrade International**	2014, FLOCERT Audit Standard Operating Procedure, Valid from 16/12/2014
	2014, FLOCERT Certification Standard Operating Procedure, Valid from 18.12.2014
	2011, Fairtrade Standard for Small Producer Organizations, Current version: 01.05.2011_v1.2
	2011, Fairtrade Standard for Coffee for Small Producer Organizations, Current version: 01.04.2011
**Fairtrade USA**	2014, Fair Trade USA: Guidelines for the Implementation and Interpretation of the Independent Smallholders Standard, September 2014
	2014, Fair Trade USA Farm Workers Standard: Guidelines for the Implementation and Interpretation of the Farm Workers Standard, April 2014
	2014, SCS Global Services Manual, Certification Manual, Fair Trade USA V2.0 English Version, Version 2-0 (June 2014)
**NESPRESSO**	2013, Generic TASQ Tool: The Tool for the Assessment of Sustainable Quality, 2013, Version 0313
**Rainforest Alliance**	2010, Sustainable Agriculture Network: Sustainable Agriculture Standard, July 2010 (version 3)
	2013, Sustainable Agriculture Network: Farm and Group Administrator Certification Policy, January 2013
	Sustainable Agriculture Network: Sustainable Agriculture Standard, For farms’ and producer groups’ crop and cattle production, 2017
**Starbucks C.A.F.E. Practices**	2014, C.A.F.E. Practices, Generic Scorecard, December 2014, Version 3.3
	2014, C.A.F.E. Practices Verifier and Inspector Operations Manual, Starbucks Coffee Company, V5.2, English Version, January 2014
**USDA**	2000, USDA National Organic Program 7 CFR Part 2015, as of September 1, 2015
**UTZ Certified**	2014, Core Code of Conduct, Version 1.0, For individual and multi-site certification
	2014, Core Code of Conduct, Version 1.0, For group and multi-group certification
	2014, Code of conduct Coffee Module, Version 1.0
	2012, UTZ Certified Certification Protocol, Version 3.0 September 2012

**Table:** 

	Name	Institution	Position
1	Frank Hicks	Forest Trends	Senior Advisor
2	Shawn Steiman	Daylight Mind	Owner and Chief Science Officer
3	Carl Cervone	Enveritas	Founder and Head of Operations
4	Andrés Guevara	Sustainable Markets Intelligence Center (CIMS)	Senior Researcher
5	Ronald Peters	Instituto del Café de Costa Rica (Icafe)	Executive Manager (Dirección Ejecutiva)
6	Dietmar Stoian	Consultative Group on International Agricultural Research (CGIAR)	Researcher
7	Jason Potts	International Institute for Sustainable Development (IISD)	Senior Associate
8	Bambi Semroc	Conservation International	Senior Strategic Advisor
9	Kraig Kraft	CRS (Catholic Relief Services) Coffeelands	Technical Advisor
10	Juan Pablo Campos	Lohas Beans	General Manager
11	Julie Craves	University of Michigan-Dearborn and Rouge River Bird Observatory	Director
12	Michiel Kuit	Agri-Logic Consultancy	Founder and co-owner
13	Daniele Giovannucci	Committee On Sustainability Assessment (COSA)	President and co-founder
14	Jan von Enden	Hanns R. Neumann Stiftung	General Manager
15	Peter Baker	Climate Edge Ltd	Director
16	Diego Pizano	Consejo Superior de Los Andes, Universidad de los Andes, Colombia	President
17	Mario Vega	ColCoffeeInsights (CCI), Café de Colombia	Commercial Manager

## Appendix A Documents on VSS

We conducted a survey among 17 highly distinguished experts in order to weigh all regulatory topics according to their relevance for sustainability improvements in global coffee production. We show the list of these experts in Appendix B.

## Appendix B Survey participants

We used a code book in order to assess the individual regulations of the different 14 VSS according to their strength. For the code book, see Appendix C.

## Appendix C Code Book

### Environmental standards

#### Protection and restoration of biodiversity

E01 Prohibitions against destroying primary forest

Regulatory elements relating to this regulation: Number of past years for which destruction is prohibited [point of reference 2016]; renaturing for destruction of primary forest done between 1999 and 2005 to compensate for negative impact; restoration of native vegetation if the farm or group of member farms have less than 15% total native vegetation cover for farms growing shade tolerant crops (non -shade tolerant 10%).

**Definitions**

Weak: Prohibitions against destroying primary forest since time of application for standard-setter.

Medium: Prohibitions against destroying primary forest for 1 to 8 years in the past.

Exception: Medium to strong (2.5 points): Prohibitions against destroying primary forest for more than 8 years in the past; restoration of native vegetation if the farm or group of member farms have less than 15% total native vegetation cover for farms growing shade tolerant crops (non -shade tolerant 10%).

Strong: Prohibitions against destroying primary forest for more than 8 years in the past; renaturing for destructions of primary forest done between 1999 and 2005 to compensate for negative impact.

E02 Prohibitions against destroying secondary forest (RFA) [natural forest (UTZ)]

Regulatory elements relating to this regulation: no destruction [no exceptions] since date of application; no destruction also for at least the past ten/eleven years (point of reference 2016]; renaturing for destructions done between 1999 and 2005 to compensate for negative impact.

**Definitions**

Weak: No destruction [no exceptions] since date of application.

Medium: Regulatory elements relating to this regulation and no destruction [no exceptions] since date of application; no destruction also for at least the past ten/eleven years (point of reference 2016].

Strong: No destruction [no exceptions] since date of application; no destruction also for at least past ten/eleven years (point of reference 2016]; renaturing for destructions done between 1999 and 2005 to compensate for negative impact.

E03 Prohibitions against destroying natural ecosystems apart from forest

Regulatory elements relating to this regulation: prohibitions against destroying high conservation areas, prohibitions against destroying any natural ecosystem, prohibitions extend to past years.

**Definitions**

Weak: Prohibitions against the destruction of high conservation areas.

Medium: Two out of the following three regulatory elements: prohibitions against destroying high conservation areas, prohibitions against destroying any natural ecosystem, prohibitions extend to past years.

Strong: Prohibitions against the destruction of high conservation areas, prohibitions against the destruction of any natural ecosystem, prohibitions extend to past years.

E04 Obligations to protect conservation areas

**Definitions**

Weak: Not applicable, no standard scored in this category.

Medium: No production occurs in protected areas unless allowed by governmental management plans.

Strong: No production occurs in protected areas (no exceptions).

E05 Obligations to establish a canopy cover on farm land (agroforestry system)

**Definitions**

Weak: Unspecified rules to promote agroforestry systems.

Exception: Weak to medium (1.5 points): At least 15% total native vegetation across the farm or group of farmers.

Medium: 40% canopy cover / two strata.

Strong: 40% canopy cover / three strata.

E06 Obligations to protect aquatic ecosystems from agrochemical drift (e.g. through the use of buffer zones)

**Definitions**

Weak: Unspecified buffer zones around water bodies.

Medium: Buffer zones and prohibitions to spray around water bodies up to 10 m for rivers and canals, up to 15 m for springs.

Strong: Very large buffer zones: more than 20 m for rivers and canals, more than 50 m for springs / General prohibition to spray agrochemicals.

E07 Prohibitions against hunting and capturing wildlife

**Definitions**

Weak: Raising awareness measures to protect wild and endangered animals.

Medium: Prohibitions against hunting endangered species.

Strong: Prohibitions against hunting, trafficking and collecting wild animals (with rare exceptions for indigenous populations).

E08 Obligations to protect terrestrial ecosystems from contamination through agrochemicals (e.g. through the use of buffer zones)

**Definitions**

Weak: Unspecified buffer zones around high conservation areas.

Medium: Large buffer zones (between 3 and 20 m around terrestrial ecosystems).

Strong: General prohibitions against spraying agrochemicals.

E09 Obligations to connect natural ecosystems

**Definitions**

Weak: Unspecified obligations to report on activities or implement plans that might include the connections of ecosystems.

Medium: Obligations to connect ecosystems with high conservation areas.

Strong: Obligations to connect all ecosystems within the farm.

E10 Obligations to separate (protect) natural ecosystems from areas of human activity and roads going through and around the farm

**Definitions**

Weak: Unspecific rules to minimize human-wildlife conflict.

Medium: Not applicable, no standard scored in this category.

Strong: Clear-cut rule to establish buffer zones between areas of human activity and buffer zones between production areas and roads.

E11 Obligations to protect endangered plant species

**Definitions**

Weak: Awareness is being raised in relation to endangered plant species.

Medium: Not applicable, no standard scored in this category.

Strong: Harvesting or otherwise removing threatened or endangered plant species is prohibited.

E12 Obligations to protect aquatic ecosystems from erosion and sedimentation

**Definitions**

Weak: Unspecified buffer zones around water bodies.

Medium: Buffer zones around water bodies up to 10 m for rivers and canals, up to 15 m for springs.

Strong: Very large buffer zones, more than 20 m for rivers and canals, more than 50 m for springs.

#### Environmentally sustainable pest and weed management

E13a Prohibitions against the use of agrochemicals

The variable is addressed by only three standard-setters by the same rule (this rule is strong by definition).

**Definition**

Strong: Strict prohibitions against the use of agrochemicals.

E13b Prohibitions against the use of most hazardous agrochemicals

**Definitions**

Weak: Not applicable, no standard scored in this category.

Medium: Only the most hazardous pesticides are prohibited (e.g. WHO 1a).

Strong: Ban of pesticides listed in international conventions and national regulations [European Union USA, Montreal Protocol, Stockholm Convention, Rotterdam Convention, PAN List of Dirty Dozen, WHO 1a and 1b]).

E14a Obligations to implement pest management practices

Regulatory elements relating to this regulation: Knowledge about products used for spraying; monitoring of pest and diseases; use of non-chemical alternatives; justified choice of chemical products; proportionality decisions [max. effectivity vs. minimum toxic impact of agrochemicals).

**Definitions**

Weak: Unspecified steps to build up an Integrated Pest Management.

Medium: Integrated Pest Management comprising 3–4 of the mentioned regulatory elements.

Strong: Integrated Pest Management comprising all of the mentioned elements.

E14b Obligations to train at least 50% of group members/workers on integrated pest management practices

The variable is addressed by only three standard-setters by the same rule (this rule is strong by definition).

**Definition**

Strong: Comprehensive trainings on implementing Integrated Pest Management practices (for list of elements: see variable E14a) are provided to at least 50% of group members/workers.

E15a Obligations to handle and apply pesticides with care in order to avoid accidents, spills and contamination of the environment

Regulatory elements relating to this regulation: Directness of the rule / scope of the rule (Elements: only trained staff handles agrochemicals; preparation and application of agrochemicals according to label and safety instructions; obligations to dispose of surplus agrochemicals in an environmentally friendly way; equipment is in good condition; empty containers need to be rinsed thoroughly and are not allowed to be used for drinking water; environmental friendly disposal of empty pesticides containers; areas for preparing agrochemicals are equipped to handle spills; emergency procedures to deal with spillages).

**Definitions**

Weak: 2–3 elements.

Medium: 2–3 elements, others elements are part of an unspecified management plan.

Strong: All or almost elements apply.

E15b Obligations to train at least 50% of group members/workers how to handle and apply pesticides in order to avoid accidents, spills and contamination of environment

The variable is addressed by only three standard-setters by the same rule (this rule is strong by definition).

**Definition**

Strong: Trainings on all measures (for list of measures see variable E15a) are provided to at least 50% of group members/workers.

E16 Obligations to store pesticides safely

Regulatory elements relating to this regulation: The farm must store only the amount of agrochemicals necessary to meet short-term needs; these products must be separated according to their biocide, toxicity and chemical formula; they must not be stored on the floor nor come into contact with absorbent materials; a Material Safety Data Sheet must be kept in the storage facility for each chemical product stored; all agrochemical containers must be washed three times before being stored for disposal or return to supplier; all agrochemical containers must maintain their original labels; the farm must take actions to return to the supplier agrochemicals that are prohibited, expired, or not legally registered, or agrochemicals that have had their licenses canceled; if the supplier will not accept them, the farm must seek safe alternatives for eliminating them.

**Definitions**

Weak: Unspecified plans to store agrochemicals.

Medium: Some rules on the list regarding storage and storage facilities apply.

Strong: All or almost all rules on storage and storage facilities apply.

E17 Obligations to restrict the use of fire for pest management

The variable is only addressed by one standard-setter (the rules of this standard-setter are strong by definition).

**Definition**

Strong: The use of fire is allowed under restrictions (Restrictions are: fire is the option of less environmental impact, use of fire is approved by competent authorities, focus on problematic areas only).

E18 Obligations to take into account resistance against pest and diseases when choosing new planting material

The variable is only addressed by one standard-setter (the rules of this standard-setter are strong by definition).

**Definition**

Strong: Resistance against pest and diseases when choosing new planting material has to be taken into account.

#### Environmentally sustainable soil management

E19 Prohibitions against the use of any inorganic fertilizers

The variable is addressed by only three standard-setters by the same rule (this rule is strong by definition).

**Definition**

Strong: Strict prohibitions against the use of inorganic fertilizers.

E20a Obligations to optimize the use of fertilizers

**Definitions**

Weak: Unspecified obligations to receive technical recommendations.

Medium: Obligations to optimize the use of organic and inorganic fertilizers according to the nutrient needs of the crop.

Strong: Use of fertilizers is based on a comprehensive fertilizer program including soil analysis.

E20b No clear obligations to optimize the use of fertilizers but obligations to provide trainings to at least 50% of group members/workers on how to optimize the use of fertilizers

**Definitions**

Weak: Not applicable, no standard scored in this category.

Medium: Trainings to fertilize according to the nutrient needs of the crop are provided to at least 50% of group members/workers.

Strong: Trainings to fertilize according to the nutrient needs of the crop, trainings on soil analysis are provided to at least 50% of group members/workers.

E21 Obligations to use organic fertilizers/natural measures to improve soil fertility

**Definitions**

Weak: Unspecified obligations to improve soil fertility.

Medium: Implementation of at least one natural measure to improve soil fertility.

Strong: Priority needs to be given to organic fertilizers, implementation of comprehensive plan to improve soil fertility by natural measures.

E22a Obligations to avoid soil erosion (e.g. through the use of ground cover)

**Definitions**

Weak: Unspecified obligations to avoid soil erosion have been started.

Medium: Implementation of a soil erosion plan.

Strong: Implementation of a soil erosion plan, plus obligations to use and expand ground cover throughout the farm.

E22b Obligations to train at least 50% of group members/workers how to avoid soil erosion

The variable is addressed by only two standard-setters by the same rule (this rule is strong by definition).

**Definitions**

Strong: Trainings on how to use ground cover and how to set up a soil erosion plan are provided to at least 50% of group members/workers.

E23 Obligations to establish new production areas based on land use capacity (climatic, soil and topographic conditions must be suitable for intensity level of the agricultural production planned)

The variable is addressed by only two standard-setters by the same rule (this rule is strong by definition).

**Definition**

Strong: Land use capacity has to be taken into account when new production areas are established.

E24 Prohibitions against burning to prepare land

The variable is addressed by only two standard-setters by the same rule (this rule is strong by definition).

**Definition**

Strong: Prohibitions against burning to prepare land.

#### Sustainable water management

E25 Prohibitions against discharging unclean wastewater into natural water bodies

**Definitions**

Weak: (no output oriented measures) Unspecified obligations not to discharge untreated water directly into water bodies.

Medium: Unspecified rules that wastewater must not have a negative impact on water quality.

Medium-strong: Comprehensive water treatment system in place, but no output-oriented measures.

Strong: Comprehensive water treatment system is place, with water analysis and corrective measures.

E26 Prohibitions against using sewage water for irrigation

The variable is addressed by only two standard-setters by the same rule (this rule is strong by definition).

**Definition**

Strong: Sewage water must not be used for irrigation.

E27 Obligations restricting the use of septic tanks for the treatment of domestic wastewaters

The variable is addressed by only one standard-setter (This rule is strong by definition).

**Definition**

Strong: Septic tanks to the treatment of domestic wastewater is only allowed under tight restrictions.

E28a Obligations to use water in a sustainable way

**Definitions**

Weak: Unspecified rules to take some measures to conserve water.

Medium: Measures to use water efficiently are in place (best available technologies, water needs are determined, water sources are mapped, used water is documented).

Strong: Measures to use water efficiently are in place, water is harvested from sustainable sources (rain harvesting), water re-circulation systems are in place.

E28b Obligations to provide training to at least 50% of group members/workers on how to use water sustainably

The variable is addressed by only two standard-setters by the same rule (this rule is strong by definition).

**Definition**

Strong: Trainings how to use water efficiently take place are provided to at least 50% of group members/workers.

#### Sustainable waste management

E29 Obligations to reduce waste

**Definitions**

Weak: Organic waste is recycled.

Medium: Non-hazardous waste is reused or recycled, organic waste is reused.

Strong: Waste management plan is in place for all types of waste.

E30 Obligations to dispose of waste in a way that reduces the risks of environmental contamination (e.g. safe waste disposals, prohibitions of burning waste)

**Definitions**

Weak: Hazardous waste (only) is safely disposed.

Medium: Organic, non-hazardous and hazardous waste is safely disposed, unspecific rules to regulate burning of waste.

Strong: Organic, non-hazardous and hazardous waste are safely disposed, strict rules to regulate burning of waste, restrictions to transfer waste to third persons.

#### Sustainable use of energy

E31 Obligations to use energy efficiently

There is no variance between the different standard-setters. Therefore, it is only measured whether a standard-setter contains obligations to use energy efficiently or not. The existence of such a rule is by definition strong.

**Definition**

Strong: Measures are taken to use energy more efficiently.

E32 Obligations to use renewable energy if economically feasible

Measure: The variable is addressed by only two standard-setters by the same rule (this rule is strong by definition).

**Definition**

Strong: Usage of renewable energy if economically feasible.

#### Genetically Modified Organisms (GMOs)

E33a Prohibitions against GMOs

There is no variance between the different standard-setters. Therefore, it is only measured whether a standard-setter prohibits GMOs or not. The existence of such a rule is by definition strong.

**Definition**

Strong: Prohibitions against GMOs.

E33b Obligations to communicate the use of GMOs to standard-setting body

The variable is addressed by only two standard-setters by the same rule (this rule is strong by definition).

**Definition**

Strong: The use of GMOs has to be communicated to the standard-setting body.

### Social standards

#### Employment conditions

S01 Obligations to pay equal or greater than minimum wage

**Definitions**

Weak: Not applicable, no standard scored in this category.

Medium: Minimum wages (national or industry branch) must be paid.

Strong: Payments must increase above minimum wage or living wage must be paid.

S02 Obligations to respect wages negotiated in collective bargaining agreements

There is no variance between the different standard-setters. Therefore, it is only measured whether a standard-setter contains such a rule or not. The existence of such a rule is strong by definition.

**Definition**

Strong: Wages must be paid according to collective bargaining negotiations, if they exist.

S03 Obligations to establish a reliable and transparent payment system

Regulatory elements relating to this regulation: Regular payment; written pay slip; prohibitions against using wage deductions for disciplinary purposes; prohibitions to ask for money for employment.

**Definitions**

Weak: Only one of the elements.

Medium: Two elements.

Strong: Three to four elements.

S04 Obligations to provide workers with legally binding written contracts

Regulatory elements relating to this regulation: regulation includes all workers employed for more than three months / permanent workers only.

**Definitions**

Weak: Not applicable, no standard scored in this category.

Medium: Permanent workers.

Strong: All workers employed for more than three months.

S05 Obligations to allow labor contractors only when they can demonstrate compliance with the requirements of a standard-setter

There is no variance between the different standard-setters. Therefore, it is only measured whether a standard-setter contains such a rule or not. The existence of such a rule is strong by definition.

**Definition**

Strong: Labor contractors are only allowed when they can demonstrate compliance with the requirements of a standard-setter.

S06 Obligations to take efforts to avoid time-contracts and to employ workers on a permanent basis

There is no variance between the different standard-setters. Therefore, it is only measured whether a standard-setter contains such a rule or not. The existence of such a rule is strong by definition.

**Definition**

Strong: Where possible the certification holder and the members of its organization must assign all permanent work to regular workers.

S07 Obligations to restrict working hours and grant vacation

Regulatory elements relating to this regulation: working hours do not exceed 48 h per week; at least one day off after 6 days of work; not more than 12 h overtime per week; breaks at least after 5 h for 30 min; working hours are recorded individually.

**Definitions**

Weak: Not applicable, no standard scored in this category.

Medium: None or almost none of the elements is explicitly mentioned but reference is made that working hours comply with national and international conventions.

Strong: All or almost all of the elements apply.

S08 Obligations to grant maternity leave

Regulatory elements relating to this regulation: 12 weeks of maternity leave; 8 weeks paid maternity leave; right to return to the same position.

**Definitions**

Weak: Not applicable, no standard scored in this category.

Medium: 12 weeks of maternity leave, right to return to same position, but not 8 weeks of payment.

Strong: All elements.

S09 Obligations grant decent housing for workers living on-site

There is no variance between the different standard-setters. Therefore, it is only measured whether a standard-setter contains such a rule or not. The existence of such a rule is strong by definition.

**Definition**

Strong: Workers living on-site have clean and safe living quarters.

S10 Obligations to establish day care facilities for children living on the farm

The variable is addressed by only two standard-setters by the same rule (this rule is strong by definition).

**Definition**

Strong: Workers living on-site have access to convenient and affordable day care services for their children.

S11 Prohibitions against making workers dependent on stores or services operated by the employer

The variable is addressed by only two standard-setters by the same rule (this rule is strong by definition).

**Definition**

Strong: Workers are not compelled to make use of stores or services operated by the employer or in connection to the company. Where access to other stores or services is limited or not possible due to location, the employer provides goods and services at fair and reasonable prices.

S12 Obligations to guarantee access to education for school age children living on the farm

Regulatory elements relating to this regulation: Children must go school; if access to schools cannot be guaranteed under reasonable conditions the farm/group must provide adequate schooling; work does not jeopardize schooling.

**Definitions**

Weak: Measures are taken to implement the above mentioned elements.

Medium: Children must go school.

Strong: All elements are fulfilled.

S13 Obligations to grant farm workers the rights to establish and join worker׳s organizations (freedom of association and collective bargaining)

There is no variance between the different standard-setters. Therefore, it is only measured whether a standard-setter contains such a rule or not. The existence of such a rule is strong by definition.

**Definition**

Strong: Workers can freely join worker׳s organizations without any negative consequences, workers are effectively informed about their rights to establish and join worker׳s organizations.

#### Non-discrimination

S14 Prohibitions against the discrimination of workers

All standard-setters contain strict prohibitions of discrimination; exceptions are USDA Organic, EU Organic and Bird Friendly (rule is strict by definition).

**Definition**

Strong: Prohibitions against the discrimination of workers.

S15 Prohibitions against corporal punishment, mental or physical coercion and verbal abuse

All standard-setters hold that workers are not subject to corporal punishment, sexual harassment, oppression, coercion or any other kind of mental or physical abuse or intimidation at the workplace; exceptions are USDA Organic, EU Organic and Bird Friendly (rule is strong by definition).

**Definition**

Strong: Prohibitions against corporal punishment, mental or physical coercion and verbal abuse.

S16 Prohibitions against sexual harassment

All standard-setters prohibit sexual harassment; exceptions are USDA Organic, EU Organic and Bird Friendly (rule is strong by definition).

**Definition**

Strong: Prohibitions against sexual harassment.

S17 Obligations to train workers

There is no variance between the different standard-setters. Therefore, it is only measured whether a standard-setter contains such a rule or not. The existence of such a rule is strong by definition.

**Definition**

Strong: Obligations to further qualify workers.

S18 Obligations to ensure that women have equal opportunities in the company

There is no variance between the different standard-setters. Therefore, it is only measured whether a standard-setter contains such a rule or not. The existence of such a rule is strong by definition.

**Definition**

Strong: A program is in place that aims to give women equal opportunities in the company.

#### Freedom from forced labor

S19 Prohibitions against forced labor

All standard-setters prohibit forced labor (rule is strong by definition).

**Definition**

Strong: Prohibitions against forced labor.

S20 Prohibitions against requiring spouses to work as a condition of employment

There is no variance between the different standard-setters. Therefore, it is only measured whether a standard-setter contains such a rule or not. The existence of such a rule is strong by definition.

**Definition**

Strong: It is not allowed to require spouses to work.

S21 Prohibitions against hiring minors under the age of 14/15

All standard-setters contain this rule (rule is strong by definition).

**Definition**

Strong: Prohibitions against hiring minors under the age of 14/15.

S22 Obligations to limit the work of children helping in family farming and family harvesting

There is no variance between the different standard-setters. Therefore, it is only measured whether a standard-setter contains such a rule or not. The existence of such a rule is strong by definition.

**Definition**

Strong: Children are allowed to help their families on the farm, children are allowed to help their families (employed families) harvesting coffee, work does not interfere with their schooling obligations, work does not exceed two hours per day, work is light.

S23 Obligations to set up remediation policies to ensure that children do no not enter into worse forms of child labor (when no longer allowed to work on the farm according to the standard-setter)

There is no variance between the different standard-setters. Therefore, it is only measured whether a standard-setter contains such a rule or not. The existence of such a rule is strong by definition.

**Definition**

Strong: Remediation policies are in place to make sure that children do not enter worse forms of child labor.

#### Community relations (land-use rights)

S24 Obligations to have a legitimate right to land use (prohibition of forced eviction, no land tenure disputes with local population)

There is no variance between the different standard-setters. Therefore, it is only measured whether a standard-setter contains such a rule or not. The existence of such a rule is strong by definition.

**Definition**

Strong: Farm must either show the appropriate legal document, or it can demonstrate that significant disputes with local communities are absent, there is consensus about land use.

#### Occupational health and safety

S25 Obligations to keep workplaces safe

Regulatory elements relating to this regulation: warning signs; requirements regarding the design and construction of workplaces: corridors and storage areas on the floor of the storage facilities must be well marked; there must be a free space of at least 30 cm between the wall and the stored materials; the storage facilities must have shelving and platforms for storing equipment made from non-absorbent materials for storing liquid products; there must be enough natural light to allow visibility during the day in the absence of electricity; there must be enough natural ventilation to prevent the accumulation of odors and vapors; the emergency exits must be clearly marked and unobstructed; in the box and packaging assembly areas, the continuous noise level must not exceed 85 dB; the box and packaging assembly areas must have at least two meters of free space for each assigned worker; the farm must have packing material (cardboard boxes, plastic and other materials) storage and assembly areas constructed from impermeable and non-flammable materials.

**Definitions**

Weak: Unspecified plans to install a safety program or requirement of warning sign only.

Medium: Clear warning signs, a few (2–3) design and construction requirements are fulfilled.

Strong: All elements are fulfilled.

S26 Obligations to keep machinery safe

There is no variance between the different standard-setters. Therefore, it is only measured whether a standard-setter contains such a rule or not. The existence of such a rule is strong by definition.

**Definition**

Strong: Clear obligations that machinery is safe.

S27 Obligations to keep spraying equipment in good order

Regulatory elements relating to this regulation: spraying equipment is safe, spraying equipment is calibrated.

**Definitions**

Weak: Unspecified requirements regarding the existence of a health and safety system.

Medium: One element.

Strong: All elements.

S28 Obligations to provide drinking water to workers

There is no variance between the different standard-setters. Therefore, it is only measured whether a standard-setter contains such a rule or not. The existence of such a rule is strong by definition.

**Definition**

Strong: Workers have access to clean drinking water.

S29 Obligations to use Personal Protective Equipment (PPE) for application of agrochemicals

Regulatory elements relating to this regulation: the use of PPE is obligatory for workers; the use of PPE is obligatory for all persons applying agrochemicals [applies only to group standards].

**Definitions**

Weak: Unspecified requirements regarding the existence of a health and safety system.

Medium: The use of PPE is obligatory for workers.

Strong: The use of PPE is obligatory for all persons applying agrochemicals.

S30 Obligations to provide occupational health and safety trainings on handling pesticides

**Definitions**

Weak: Not applicable, no standard scored in this category.

Medium: Applies to workers only.

Strong: Applies to all persons.

S31 Obligations to provide first aid and medical care

Regulatory elements relating to this regulation: access to first aid by trained persons; first aid boxes are provided; in case of group standards, rule applies to workers only or all persons.

**Definitions**

Weak: Unspecified requirements regarding the existence of a health and safety system.

Medium: First aid boxes are provided.

Strong: Both elements.

S32 Obligations to provide medical check-ups to workers handling any potentially hazardous work

There is no variance between the different standard-setters. Therefore, it is only measured whether a standard-setter contains such a rule or not. The existence of such a rule is strong by definition.

**Definition**

Strong: Regular check-ups are obligatory.

S33 Obligations to provide sanitary facilities

There is no variance between the different standard-setters. Therefore, it is only measured whether a standard-setter contains such a rule or not. The existence of such a rule is strong by definition.

**Definition**

Strong: Obligations to provide appropriate sanitary facilities at workplaces.

### Economic standards

#### Yield optimization

W01 Obligations requiring the management of a cooperative or estate to receive consulting on Good Agricultural Practices (GAP)

**Definition**

Weak: Not applicable, no standard scored in this category.

Medium: Advice on at least one aspect of GAP.

Strong: Comprehensive advice on all aspects of GAP.

W02 Obligations requiring coffee producers to implement GAP

Regulatory elements relating to this regulation: implementation of procedures to systematically improve soil quality / implementation of GAP in the field of soil improvement; implementation of GAP in the field of harvesting.

**Definition**

Weak: Not applicable, no standard scored in this category.

Medium: At least one element.

Strong: All elements.

W03 Obligations requiring coffee producers to implement good practices for storing, handling and processing coffee

The variable is addressed by only two standard-setters by the same rule (this rule is strong by definition).

**Definition**

Strong: Comprehensive obligations to implement good practices for storing, handling and processing coffee.

W04 Obligations requiring the management of a cooperative or estate to invest portions of premiums to increase productivity and quality of coffee production

The variable is addressed by only two standard-setters by the same rule (this rule is strong by definition).

**Definition**

Strong: At least 5cts of the Fairtrade premium must be invested in the improvement of productivity and/or quality of Fairtrade coffee.

#### Premiums and financing

W05 Obligations requiring coffee buyers to grant a minimum price guarantee to coffee producers

The variable is addressed by only two standard-setters by the same rule (this rule is strong by definition).

**Definition**

Strong: Minimum price guarantee exists.

W06a Obligations requiring coffee buyers to grant coffee producers premium payments that are regulated by the market

There is no variance between the different standard-setters. Therefore, it is only measured whether a standard-setter contains such a rule or not. The existence of such a rule is strong by definition.

**Definition**

Strong: Obligations requiring coffee buyers to grant coffee producers premium payments that are regulated by the market.

W06b Obligations requiring coffee buyers to grant mandatory premiums to coffee producers set by the standard-setting body

Applies only to all Fairtrade International and Fair Trade USA standards (the rule is strong by definition).

**Definition**

Strong: Obligations requiring coffee buyers to grant mandatory premiums to coffee producers set by the standard-setting body.

W07 Obligations requiring the management of a cooperative or estate to use premiums according to communal development or work plans approved by an inclusive general assembly

Applies only to all Fairtrade International and Fair Trade USA standards (rule is strong by definition).

**Definition**

Strong: Obligations requiring the management of a cooperative or estate to use premiums according to communal development or work plans approved by an inclusive general assembly.

W08 Obligations requiring coffee buyers to pre-finance coffee production

Applies only to all Fairtrade International standards (rule is strong by definition).

**Definition**

Strong: Obligations requiring coffee buyers to pre-finance coffee production.

#### Smallholder focus

W09 Obligations requiring Voluntary Sustainability Standards (VSS) to certify only self-administered smallholder producer organizations

Applies only to all Fairtrade International standards (rule is strong by definition).

**Definition**

Strong: Obligations requiring VSS to certify only self-administered smallholder producer organizations.

### Enforcement

#### Control of producers

C01 Obligations requiring the auditor to select a representative farm sample that is subjected to field audits

**Definitions**

Weak: Sample calculation represents less than the square root of all farms.

Medium: Sample calculation represents the square root of all farms.

Strong: Sample calculation represents more than the square root of all farms or square root plus risk analysis.

C02 Obligations requiring the auditor to re-certify certification holders according to pre-determined audit cycles (assumption: the shorter the audit cycle the stricter the VSS)

**Definitions**

Weak: Three of more years before full re-audit, no external controls in the years between.

Medium: Three or more years before full re-audit, less intense external controls in the year between.

Strong: Annual re-audit, or full external audits in the years between.

C03 Obligations requiring the auditor to conduct unannounced audits

Regulatory elements relating to this regulation: Comprehensiveness of unannounced audits (elements: cost carried by standard-setting program not auditors; unannounced audits for at least 10% of total number of audited units are obligatory; no warning period; selection of the units that receive unannounced audits according to predefined risk criteria not at discretion of auditors).

**Definition**

Weak: Unspecified mentioning that unannounced audits might be possible.

Medium: At least two of the elements.

Strong: Three or four elements.

C04 Obligations requiring the auditor to conduct interviews with workers

Regulatory elements relating to this regulation: Precision of the rule / scope of the rule (elements: confidentiality; representative selection of interviewees including workers from different levels and groups; specification of a significant number of interviews, e.g. 50 workers/15 interviews)

**Definitions**

Weak: Unspecified mentioning that interviews with workers are part of the auditing process.

Medium: At least one element.

Strong: More than one element.

#### Control of auditors

C05 Obligation requiring auditors to be ISO certified

All standard setters accept only auditors that are certified by ISO (rule is strong by definition).

**Definition**

Strong: Obligation requiring auditors to be ISO certified.

C06 Obligations requiring auditors to accept shadow audits though the standard setter (in order to evaluate audit quality)

**Definition**

Strong: Obligations requiring auditors to accept shadow audits though the standard setter (in order to evaluate audit quality).

C07a Obligations that standard owned auditor is the only auditor allowed to conduct producer audits

**Definition**

Strong: Obligations that standard owned auditor is the only auditor allowed to conduct producer audits.

C07b Obligations that one independent auditor determined by the standard setter must conduct all audits

**Definition**

Strong: Obligations that one independent auditor determined by the standard setter must conduct all audits.

C07c Obligations requiring the standard setter to provide a list of independent auditors(coffee producers can choose auditors from this list)

**Definition**

Strong: Obligations requiring the standard setter to provide a list of independent auditors. Coffee producers can choose auditors from this list.
