# Supplementary material for: Indicators to compare and assess the institutional strength of voluntary sustainability standards in the global coffee industry
Source: Data Brief. 2018 May 16;19:570–85. doi: 10.1016/j.dib.2018.05.048 (PMC5997897; doi:10.1016/j.dib.2018.05.048)
Supplement: Supplementary file 1 — Supplementary material [file mmc1.docx]

The authors certify that they have NO affiliations with or involvement in any organization or entity with any financial interest (such as honoraria; educational grants; participation in speakers’ bureaus; membership, employment, consultancies, stock ownership, or other equity interest; and expert testimony or patent-licensing arrangements), or non-financial interest (such as personal or professional relationships, affiliations, knowledge or beliefs) in the subject matter or materials discussed in this manuscript.
