# Supplementary material for: Indicators to compare and assess the institutional strength of voluntary sustainability standards in the global coffee industry
Source: Data Brief. 2018 May 16;19:570–85. doi: 10.1016/j.dib.2018.05.048 (PMC5997897; doi:10.1016/j.dib.2018.05.048)
Supplement: Supplementary file 2 — Supplementary material [file mmc2.docx]

**Table 1: Indicator results**

|  | Weights | FLO/EU Organic^[[1]](#footnote-1)^ | UTZ | Fair Trade USA | Rainforest Alliance | UTZ (SH) | Rainforest Alliance (SH) | Rainforest Alliance (2010) | Fairtrade International | Fair Trade USA (SH) | Bird Friendly | Nespresso AAA | 4C | C.A.F.E Practices | USDA Organic | EU Organic |
| --- | --- | --- | --- | --- | --- | --- | --- | --- | --- | --- | --- | --- | --- | --- | --- | --- |
| **Environmental indicators** | | | | | | | | | | | | | | | | |
| E01 Prohibitions against destroying primary forest | 0.8 | 0.8 | 1.6 | 1.6 | 2.0 | 1.6 | 2.0 | 2.4 | 0.8 | 0.8 | 2.4 | 2.4 | 2.4 | 2.4 | 0.0 | 0.0 |
| E02 Prohibitions against destroying secondary forest (RFA) [natural forest (UTZ)] | 0.8 | 0.0 | 0.8 | 0.0 | 1.9 | 0.8 | 1.9 | 2.3 | 0.0 | 0.0 | 2.3 | 1.6 | 0.0 | 1.6 | 0.0 | 0.0 |
| E03 Prohibitions against destroying natural ecosystems apart from forest | 0.8 | 0.8 | 0.0 | 0.8 | 1.9 | 0.0 | 1.9 | 2.3 | 0.8 | 0.8 | 0.0 | 1.6 | 0.0 | 0.0 | 0.0 | 0.0 |
| E04 Obligations to protect conservation areas | 0.7 | 1.4 | 1.4 | 1.4 | 2.1 | 1.4 | 2.1 | 1.7 | 1.4 | 0.7 | 0.0 | 0.0 | 1.4 | 0.0 | 0.0 | 0.0 |
| E05 Obligations to establish a canopy cover on farm land (agroforestry system) | 0.5 | 0.5 | 0.5 | 0.5 | 0.3 | 0.5 | 0.3 | 0.8 | 0.5 | 0.5 | 1.5 | 0.5 | 0.0 | 0.0 | 0.0 | 0.0 |
| E06 Obligations to protect aquatic ecosystems from agrochemical drift (e.g. through the use of buffer zones) | 0.8 | 2.3 | 1.6 | 0.8 | 0.8 | 1.6 | 0.8 | 1.9 | 0.0 | 0.0 | 2.3 | 0.0 | 0.0 | 0.0 | 2.3 | 2.3 |
| E07 Prohibitions against hunting and capturing wildlife | 0.5 | 0.0 | 1.0 | 1.0 | 1.0 | 1.0 | 1.0 | 1.4 | 0.0 | 1.0 | 0.5 | 1.4 | 1.0 | 0.0 | 0.0 | 0.0 |
| E08 Obligations to protect terrestrial ecosystems from contamination through agrochemicals (e.g. through the use of buffer zones) | 0.7 | 2.1 | 0.7 | 0.7 | 1.1 | 0.7 | 1.1 | 1.1 | 0.7 | 0.7 | 2.1 | 0.0 | 0.0 | 0.0 | 2.1 | 2.1 |
| E09 Obligations to connect natural ecosystems | 0.5 | 0.5 | 0.0 | 1.1 | 0.5 | 0.0 | 0.5 | 1.3 | 0.5 | 0.5 | 1.6 | 0.0 | 0.0 | 0.0 | 0.0 | 0.0 |
| E10 Obligations to separate (protect) natural ecosystems from areas of human activity and roads going through and around the farm | 0.5 | 0.0 | 0.0 | 0.0 | 0.0 | 0.0 | 0.0 | 1.1 | 0.0 | 0.0 | 0.0 | 0.0 | 0.0 | 0.0 | 0.0 | 0.0 |
| E11 Obligations to protect endangered plant species | 0.6 | 0.0 | 1.7 | 0.0 | 1.6 | 1.7 | 1.6 | 1.4 | 0.0 | 0.0 | 0.0 | 1.7 | 0.0 | 0.0 | 0.0 | 0.0 |
| E12 Obligations to protect aquatic ecosystems from erosion and sedimentation | 0.7 | 0.7 | 1.5 | 0.7 | 0.7 | 1.5 | 0.7 | 1.7 | 0.7 | 0.7 | 2.2 | 0.0 | 0.0 | 0.0 | 0.0 | 0.0 |
| E13a^[[2]](#footnote-2)^ Prohibitions against the use of agrochemicals | 0.2 | 0.7 | 0.0 | 0.0 | 0.0 | 0.0 | 0.0 | 0.0 | 0.0 | 0.0 | 0.7 | 0.0 | 0.0 | 0.0 | 0.7 | 0.7 |
| E13b Prohibitions against the use of most hazardous agrochemicals | 0.8 | 1.7 | 2.5 | 2.5 | 2.5 | 2.5 | 2.5 | 2.5 | 1.7 | 2.5 |  | 2.5 | 1.7 | 2.5 |  |  |
| E14a Obligations to implement pest management practices | 0.7 | 2.1 (1.4 +  1.2) | 2.1 | 2.1 (1.4 +  1.2) | 1.3 | 2.1 | 1.3 | 1.7 | 2.1  (1.4 + 1.2) | 2.1 (1.4 +  1.2) |  | 0.0 | 0.0 | 0.0 |  |  |
| E14b Obligations to train at least 50% of group members/workers on integrated pest management practices | 0.4 |  | 0.0 |  | 0.0 | 0.0 | 0.0 | 0.0 |  |  |  | 0.0 | 0.0 | 0.0 |  |  |
| E15a Obligations to handle and apply pesticides with care in order to avoid accidents, spills and contamination of environment | 0.9 | 0.9 | 2.6 | 2.6 (1.8 +  1.1) | 2.4 | 2.6 | 2.4 | 2.1 | 0.9 | 2.6 (1.8 +  1.1) |  | 0.0 | 0.9 | 0.0 |  |  |
| E15b Obligations to train at least 50% of group members/workers on how to handle and apply pesticides in order to avoid accidents, spills and contamination of environment | 0.4 | 1.1 | 0.0 |  | 0.0 | 0.0 | 0.0 | 0.0 | 1.1 |  |  | 0.0 | 0.0 | 0.0 |  |  |
| E16 Obligations to store pesticides safely | 0.8 | 1.6 | 2.4 | 1.6 | 1.4 | 0.8 | 1.4 | 1.9 | 1.6 | 1.6 |  | 0.0 | 0.8 | 0.0 |  |  |
| E17 Obligations to restrict the use of fire for pest management | 0.6 | 0.0 | 0.0 | 0.0 | 1.5 | 0.0 | 1.5 | 1.3 | 0.0 | 0.0 |  | 0.0 | 0.0 | 0.0 | 0.0 | 0.0 |
| E18 Obligations to take into account resistance against pest and diseases when choosing new planting material | 0.7 | 0.0 | 2.0 | 0.0 | 0.0 | 2.0 | 0.0 | 0.0 | 0.0 | 0.0 |  | 0.0 | 0.0 | 0.0 |  |  |
| E19 Prohibitions against the use of any inorganic fertilizers | 0.2 | 0.5 | 0.0 | 0.0 | 0.0 | 0.0 | 0.0 | 0.0 | 0.0 | 0.0 | 0.5 | 0.0 | 0.0 | 0.0 | 0.5 | 0.5 |
| E20a Obligations to optimize the use of fertilizers | 0.8 | 0.0 | 2.3 | 1.6 | 0.7 | 1.6 | 0.7 | 2.3 | 0.0 | 0.0 |  | 0.0 | 0.8 | 0.0 |  |  |
| E20b Obligations to provide trainings of group members/workers on how to optimize the use of fertilizers | 0.5 | 1.4 | 0.0 | 0.0 | 0.0 | 0.0 | 0.0 | 0.0 | 1.4 | 0.9 |  | 0.0 | 0.0 | 0.0 |  |  |
| E21 Obligations to use organic fertilizers/natural measures to improve soil fertility | 0.6 | 1.7 | 1.7 | 1.1 | 1.0 | 1.1 | 1.0 | 1.3 | 1.1 | 0.6 | 1.7 | 0.0 | 0.0 | 0.0 | 1.7 | 1.7 |
| E22a Obligations to avoid soil erosion (e.g. through the use of ground cover) | 0.8 | 2.3 (1.5 +  1.4) | 1.5 | 0.8 | 1.4 | 1.5 | 1.4 | 1.8 | 0.0 | 0.8 | 1.5 | 0.0 | 0.8 | 0.0 | 1.5 | 1.5 |
| E22b Obligations to train at least 50% of group members/workers on how to avoid soil erosion | 0.5 |  | 0.0 | 0.0 | 0.0 | 0.0 | 0.0 | 0.0 | 1.4 | 0.9 |  | 0.0 | 0.0 | 0.0 | 0.0 | 0.0 |
| E23 Obligations to establish new production areas based on land use capacity (climatic, soil and topographic conditions must be suitable for intensity level of the agricultural production planned) | 0.6 | 0.0 | 0.0 | 0.0 | 1.8 | 0.0 | 1.8 | 1.4 | 0.0 | 0.0 |  | 1.8 | 0.0 | 0.0 | 0.0 | 0.0 |
| E24 Prohibitions against burning to prepare land | 0.6 | 0.0 | 0.0 | 0.0 | 1.6 | 0.0 | 1.6 | 1.4 | 0.0 | 0.0 |  | 1.8 | 0.0 | 0.0 | 0.0 | 0.0 |
| E25 Prohibitions against discharging uncleaned wastewater into natural water bodies | 0.9 | 0.9 | 2.8 | 1.9 | 2.3 | 2.8 | 2.3 | 2.2 | 0.9 | 0.9 | 0.9 | 1.9 | 0.9 | 0.0 | 0.0 | 0.0 |
| E26 Prohibitions against using sewage water for irrigation | 0.8 | 0.0 | 2.3 | 2.3 | 2.3 | 0.0 | 2.3 | 0.0 | 0.0 | 0.0 | 0.0 | 0.0 | 0.0 | 0.0 | 0.0 | 0.0 |
| E27 Obligations restricting the use of septic tanks to the treatment of domestic wastewaters | 0.6 | 0.0 | 0.0 | 0.0 | 1.1 | 0.0 | 1.1 | 1.4 | 0.0 | 0.0 | 0.0 | 0.0 | 0.0 | 0.0 | 0.0 | 0.0 |
| E28a Obligations to use water in a sustainable way | 0.8 | 0.0 | 1.7 | 0.8 | 1.5 | 1.7 | 1.5 | 2.0 | 0.0 | 0.8 | 2.5 | 0.0 | 0.8 | 0.0 | 0.0 | 0.0 |
| E28b Obligations to train at least 50% of group members/workers on how to use water sustainably | 0.6 | 1.1 | 0.0 | 0.0 | 0.0 | 0.0 | 0.0 | 0.0 | 1.1 | 1.7 | 0.0 | 0.0 | 0.0 | 0.0 | 0.0 | 0.0 |
| E29 Obligations to reduce waste | 1.0 | 0.0 | 2.0 | 1.0 | 2.7 | 2.0 | 2.7 | 2.4 | 0.0 | 1.0 | 1.0 | 0.0 | 0.0 | 0.0 | 0.0 | 0.0 |
| E30 Obligations to dispose waste in a way that reduces the risks of environmental contamination | 1.0 | 2.0 | 2.0 | 2.0 | 1.8 | 2.0 | 1.8 | 2.4 | 2.0 | 2.0 | 1.0 | 0.0 | 1.0 | 0.0 | 0.0 | 0.0 |
| 31 Obligations to use energy efficiently | 0.6 | 0.0 | 1.7 | 1.7 | 1.0 | 0.0 | 1.0 | 1.3 | 0.0 | 1.7 | 0.0 | 0.0 | 1.7 | 0.0 | 0.0 | 0.0 |
| 32 Obligations to use renewable energy if economically feasible | 0.5 | 0.0 | 1.6 | 1.6 | 0.9 | 0.0 | 0.9 | 0.0 | 0.0 | 0.0 | 0.0 | 0.0 | 0.0 | 0.0 | 0.0 | 0.0 |
| 33a Prohibitions against using GMOs | 1.0 | 3.0 | 0.0 | 3.0 | 3.0 | 0.0 | 3.0 | 2.4 | 3.0 | 3.0 | 3.0 | 3.0 | 3.0 | 0.0 | 3.0 | 3.0 |
| 33b Obligations to communicate the use of GMOs to standard-setting body | 1.0 | 0.0 | 3.0 | 0.0 | 0.0 | 3.0 | 0.0 | 0.0 | 0.0 | 0.0 | 0.0 | 0.0 | 0.0 | 0.0 | 0.0 | 0.0 |
| **Social indicators** | | | | | | | | | | | | | | | | |
| S01 Obligations to pay equal or greater than minimum wage | 0.8 | 1.6 | 2.4 | 1.6 | 2.4 | 1.6 | 2.4 | 1.6 | 1.6 | 1.6 | 1.6 | 1.6 | 1.6 | 1.6 | 0.0 | 0.0 |
| S02 Obligations to respect wages negotiated in collective bargaining agreements | 0.8 | 2.5 | 2.5 | 2.5 | 2.5 | 2.5 | 2.5 | 2.5 | 2.5 | 2.5 | 0.0 | 0.0 | 0.0 | 0.0 | 0.0 | 0.0 |
| S03 Obligations to establish a reliable and transparent payment system | 0.8 | 1.7 | 2.5 | 2.5 | 2.2 | 2.5 | 2.2 | 2.0 | 1.7 | 0.8 | 0.0 | 0.0 | 0.0 | 0.0 | 0.0 | 0.0 |
| S04 Obligations to provide workers with legally binding written contracts | 0.6 | 0.6 | 1.9 | 1.9 | 0.0 | 1.9 | 0.0 | 0.0 | 0.6 | 0.0 | 0.0 | 0.0 | 0.0 | 0.0 | 0.0 | 0.0 |
| S05 Obligations to allow labor contractors only when they can demonstrate compliance with the certification requirements | 0.7 | 2.0 | 0.0 | 2.0 | 2.0 | 0.0 | 2.0 | 1.6 | 2.0 | 0.0 | 0.0 | 0.0 | 0.0 | 2.0 | 0.0 | 0.0 |
| S06 Obligations to take efforts to avoid time-contracts and to employ workers on a permanent basis | 0.4 | 1.3 | 0.0 | 1.3 | 1.3 | 0.0 | 1.3 | 0.0 | 1.3 | 1.3 | 0.0 | 0.0 | 0.0 | 0.0 | 0.0 | 0.0 |
| S07 Obligations to restrict working hours and grant vacation | 0.5 | 0.0 | 1.4 | 0.0 | 1.4 | 0.0 | 0.5 | 1.1 | 0.0 | 0.0 | 0.0 | 0.0 | 1.0 | 0.0 | 0.0 | 0.0 |
| S08 Obligations to grant maternity leave | 0.7 | 0.0 | 1.3 | 2.0 | 1.2 | 1.3 | 0.0 | 0.0 | 0.0 | 1.3 | 0.0 | 0.0 | 0.0 | 0.0 | 0.0 | 0.0 |
| S09 Obligations to grant decent housing for workers living on-site | 0.8 | 0.0 | 2.5 | 2.5 | 2.2 | 0.0 | 2.2 | 2.0 | 0.0 | 0.0 | 0.0 | 0.0 | 2.5 | 0.0 | 0.0 | 0.0 |
| S10 Obligations to establish day care facilities for children living on the farm | 0.7 | 0.0 | 2.0 | 2.0 | 0.0 | 0.0 | 0.0 | 0.0 | 0.0 | 0.0 | 0.0 | 0.0 | 0.0 | 0.0 | 0.0 | 0.0 |
| S11 Prohibitions against making workers dependent on stores or services operated by the employer | 0.7 | 0.0 | 0.0 | 1.4 | 0.0 | 0.0 | 0.0 | 0.0 | 0.0 | 0.0 | 0.0 | 0.0 | 0.0 | 0.0 | 0.0 | 0.0 |
| S12 Obligations to guarantee access to education for school-age children | 0.8 | 0.0 | 2.3 | 1.5 | 1.4 | 0.8 | 2.0 | 1.8 | 0.0 | 0.0 | 0.0 | 0.0 | 0.0 | 1.5 | 0.0 | 0.0 |
| S13 Obligations to grant farm workers the rights to establish and join worker's organizations (freedom of association and collective bargaining) | 0.8 | 2.3 | 2.3 | 2.3 | 2.3 | 0.0 | 0.0 | 2.3 | 2.3 | 1.6 | 0.0 | 0.0 | 2.3 | 0.0 | 0.0 | 0.0 |
| S14 Prohibitions against worker discrimination | 0.9 | 2.6 | 2.6 | 2.6 | 2.6 | 2.6 | 2.6 | 2.6 | 2.6 | 2.6 | 0.0 | 2.6 | 2.6 | 2.6 | 0.0 | 0.0 |
| S15 Prohibitions against corporal punishment, mental or physical coercion and verbal abuse | 0.9 | 2.7 | 2.7 | 2.7 | 2.7 | 2.7 | 2.7 | 2.7 | 2.7 | 2.7 | 0.0 | 2.7 | 2.7 | 2.7 | 0.0 | 0.0 |
| S16 Prohibitions against sexual harassment | 0.9 | 2.6 | 2.6 | 2.6 | 2.6 | 2.6 | 2.6 | 2.6 | 2.6 | 2.6 | 0.0 | 2.6 | 2.6 | 2.6 | 0.0 | 0.0 |
| S17 Obligations to train workers | 0.8 | 0.0 | 2.3 | 2.3 | 2.1 | 0.0 | 2.1 | 0.0 | 0.0 | 0.0 | 0.0 | 0.0 | 0.0 | 0.0 | 0.0 | 0.0 |
| S18 Obligations to ensure that women have equal opportunities in the company | 0.8 | 0.0 | 2.5 | 2.5 | 2.5 | 2.5 | 2.2 | 0.0 | 0.0 | 0.0 | 0.0 | 0.0 | 0.0 | 0.0 | 0.0 | 0.0 |
| S19 Prohibitions against forced labor | 0.9 | 2.7 | 2.7 | 2.7 | 2.7 | 2.7 | 2.7 | 2.7 | 2.7 | 2.7 | 0.0 | 2.7 | 2.7 | 2.7 | 0.0 | 0.0 |
| S20 Prohibitions against requiring spouses to work as a condition of employment | 0.7 | 2.1 | 2.1 | 2.1 | 0.0 | 2.1 | 0.0 | 0.0 | 2.1 | 2.1 | 0.0 | 0.0 | 0.0 | 0.0 | 0.0 | 0.0 |
| S21 Prohibitions against hiring minors under the age of 14/15 | 0.9 | 2.6 | 2.6 | 2.6 | 2.6 | 2.6 | 2.6 | 2.6 | 2.6 | 2.6 | 0.0 | 2.6 | 2.6 | 2.6 | 0.0 | 0.0 |
| S22 Obligations to limit the work of children helping in family farming and family harvesting | 0.7 | 2.0 | 2.0 | 2.0 | 0.0 | 2.0 | 0.0 | 2.0 | 2.0 | 2.0 | 0.0 | 0.0 | 0.0 | 0.0 | 0.0 | 0.0 |
| S23 Obligations to set up remediation policies to ensure that children do not enter into worse forms of child labor (when no longer allowed to work on the farm according to the certification) | 0.8 | 2.4 | 0.0 | 2.4 | 0.0 | 0.0 | 0.0 | 0.0 | 2.4 | 2.4 | 0.0 | 0.0 | 0.0 | 0.0 | 0.0 | 0.0 |
| S24 Obligations to have a legitimate right to land use (prohibition of forced eviction, no land disputes with local population) | 0.7 | 0.0 | 2.0 | 0.0 | 2.0 | 2.0 | 2.0 | 1.6 | 0.0 | 0.0 | 0.0 | 0.0 | 2.0 | 0.0 | 0.0 | 0.0 |
| S25 Obligations to keep workplaces safe | 0.8 | 0.8 | 0.8 | 1.7 | 2.2 | 0.0 | 2.2 | 2.0 | 0.8 | 0.8 | 0.0 | 0.0 | 0.8 | 0.0 | 0.0 | 0.0 |
| S26 Obligations to keep machinery is safe | 0.8 | 1.7 | 2.5 | 2.5 | 1.7 | 2.5 | 1.7 | 0.0 | 1.7 | 2.5 | 0.0 | 0.0 | 2.5 | 0.0 | 0.0 | 0.0 |
| S27 Obligations to keep spraying equipment is in good order | 0.9 | 1.7 | 2.6 | 1.7 | 2.6 | 0.0 | 2.6 | 2.0 | 1.7 | 1.7 | 0.0 | 0.0 | 0.9 | 0.0 | 0.0 | 0.0 |
| S28 Obligations to provide drinking water to workers | 0.9 | 2.6 | 2.6 | 2.6 | 2.6 | 0.0 | 2.6 | 2.1 | 2.6 | 2.6 | 0.0 | 0.0 | 2.6 | 0.0 | 0.0 | 0.0 |
| S29 Obligations to use Personal Protective Equipment for application of agrochemicals | 0.9 | 2.7 | 2.7 | 2.7 | 2.7 | 2.7 | 2.7 | 1.8 | 2.7 | 1.8 |  | 1.8 | 0.9 | 0.0 |  |  |
| S30 Obligations to provide occupational health and safety trainings to handle pesticides | 0.9 | 1.8 | 2.7 | 2.7 | 2.7 | 2.7 | 2.7 | 1.8 | 1.8 | 1.8 |  | 0.0 | 0.0 | 0.0 |  |  |
| S31 Obligations to provide first aid and medical care | 0.9 | 2.7 | 2.7 | 2.7 | 2.4 | 2.7 | 2.4 | 1.4 | 2.7 | 1.8 | 0.0 | 0.0 | 0.9 | 0.0 | 0.0 | 0.0 |
| S32 Obligations to provide medical check-ups to workers handling any potentially hazardous work | 0.8 | 0.0 | 2.5 | 2.5 | 2.2 | 0.0 | 2.2 | 2.0 | 0.0 | 2.5 | 0.0 | 0.0 | 0.0 | 0.0 | 0.0 | 0.0 |
| S33 Obligations to provide sanitary facilities | 0.9 | 2.6 | 2.6 | 2.6 | 2.3 | 0.0 | 2.3 | 2.0 | 2.6 | 2.6 | 0.0 | 0.0 | 0.0 | 0.0 | 0.0 | 0.0 |
| **Economic indicators** | | | | | | | | | | | | | | | | |
| W01 Obligations requiring the management of a cooperative or estate to receive consulting on Good Agricultural Practices (GAP) | 0.6 | 0.0 | 1.8 | 0.0 | 0.0 | 1.8 | 0.0 | 0.0 | 0.0 | 0.0 | 0.0 | 0.0 | 0.0 | 0.0 | 0.0 | 0.0 |
| W02 Obligations requiring coffee producers to implement GAP | 0.7 | 0.0 | 2.0 | 1.4 | 1.8 | 2.0 | 1.8 | 0.0 | 0.0 | 0.7 | 0.0 | 0.0 | 0.0 | 0.0 | 0.0 | 0.0 |
| W03 Obligations requiring coffee producers to implement good practices for storing, handling and processing coffee | 0.7 | 0.0 | 2.2 | 0.0 | 0.0 | 2.2 | 0.0 | 0.0 | 0.0 | 0.0 | 0.0 | 0.0 | 0.0 | 0.0 | 0.0 | 0.0 |
| W04 Obligations requiring the management of a cooperative or estate to invest portions of premiums to increase productivity and quality of coffee production | 0.6 | 1.9 | 0.0 | 0.0 | 0.0 | 0.0 | 0.0 | 0.0 | 1.9 | 0.0 | 0.0 | 0.0 | 0.0 | 0.0 | 0.0 | 0.0 |
| W05 Obligations requiring coffee buyers to grant a minimum price guarantee to coffee producers | 0.5 | 1.4 | 0.0 | 1.4 | 0.0 | 0.0 | 0.0 | 0.0 | 1.4 | 1.4 | 0.0 | 0.0 | 0.0 | 0.0 | 0.0 | 0.0 |
| W06a Obligations requiring coffee buyers to grant coffee producers premium payments that are regulated by the market | 0.6 | 1.9 (1.9 +  1.7) | 1.9 | 0.0 | 1.9 | 1.9 | 1.9 | 1.9 | 0.0 | 0.0 | 1.9 | 1.9 | 1.9 | 1.9 | 1.9 | 1.9 |
| W06b Obligations requiring coffee buyers to grant coffee producers mandatory premium payments set by the standard-setting body | 0.6 |  | 0.0 | 1.7 | 0.0 | 0.0 | 0.0 | 0.0 | 1.7 | 1.7 | 0.0 | 0.0 | 0.0 | 0.0 | 0.0 | 0.0 |
| W07 Obligations requiring the management of a cooperative or estate to use premiums according to communal development or work plans approved by an inclusive general assembly | 0.6 | 1.7 | 0.0 | 1.7 | 0.0 | 0.0 | 0.0 | 0.0 | 1.7 | 1.7 | 0.0 | 0.0 | 0.0 | 0.0 | 0.0 | 0.0 |
| W08 Obligations requiring coffee buyers to pre-finance coffee production | 0.5 | 1.4 | 0.0 | 0.0 | 0.0 | 0.0 | 0.0 | 0.0 | 1.4 | 0.0 | 0.0 | 0.0 | 0.0 | 0.0 | 0.0 | 0.0 |
| W09 Obligations requiring Voluntary Sustainability Standards (VSS) to certify only self-administered smallholder producer organizations | 0.3 | 0.9 | 0.0 | 0.0 | 0.0 | 0.0 | 0.0 | 0.0 | 0.9 | 0.0 | 0.0 | 0.0 | 0.0 | 0.0 | 0.0 | 0.0 |
| **Enforcement indicators** | | | | | | | | | | | | | | | | |
| C01 Obligations requiring auditors to select a representative farm sample that is subjected to field audits | 0.5 | 1.0 | 1.5 | 1.0 | 1.5 | 1.5 | 1.5 | 1.5 | 0.5 | 1.0 | 1.0 | 1.5 | 0.5 | 1.5 | 1.0 | 1.0 |
| C02 Obligations requiring auditors to re-certify certification holders according to pre-determined audit cycles (assumption: the shorter the audit cycle the stricter the VSS) | 0.5 | 1.5 | 1.5 | 1.5 | 1.5 | 1.5 | 1.5 | 1.5 | 1.5 | 1.0 | 1.5 | 1.5 | 0.5 | 1.5 | 1.5 | 1.5 |
| C03 Obligations requiring auditors to conduct unannounced audits | 0.7 | 2.1 | 2.1 | 1.4 | 1.4 | 2.1 | 1.4 | 1.4 | 0.7 | 1.4 | 2.1 | 0.0 | 0.0 | 0.0 | 2.1 | 2.1 |
| C04 Obligations requiring auditors to conduct interviews with workers | 0.7 | 2.2 | 0.0 | 1.5 | 1.5 | 0.0 | 1.5 | 1.5 | 2.2 | 1.5 | 1.5 | 1.5 | 1.5 | 1.5 | 1.5 | 1.5 |
| C05 Obligation requiring auditors to be ISO certified | 0.4 | 1.3 | 1.3 | 1.3 | 1.3 | 1.3 | 1.3 | 1.3 | 1.3 | 1.3 | 1.3 | 1.3 | 1.3 | 1.3 | 1.3 | 1.3 |
| C06 Obligations requiring auditors to accept shadow audits though the standard setter (in order to evaluate audit quality) | 0.7 | 0.0 | 2.0 | 0.0 | 0.0 | 2.0 | 0.0 | 0.0 | 0.0 | 0.0 | 0.0 | 0.0 | 0.0 | 0.0 | 0.0 | 0.0 |
| C07a Obligation that standard-owned auditor is the only auditor allowed to conduct producer audits | 0.8 | 2.3 | 0.0 | 0.0 | 2.3 | 0.0 | 2.3 | 2.3 | 2.3 | 0.0 | 0.0 | 0.0 | 0.0 | 0.0 | 0.0 | 0.0 |
| C07b Obligation that one independent auditor determined by the standard setter must conduct all audits | 1.0 | 0.0 | 0.0 | 2.9 | 0.0 | 0.0 | 0.0 | 0.0 | 0.0 | 2.9 | 0.0 | 2.9 | 0.0 | 2.9 | 0.0 | 0.0 |
| C07c Obligation requiring the standard setter to provide a list of independent auditors (coffee producers can choose auditors from this list) | 0.5 | 1.5 | 1.5 | 0.0 | 0.0 | 1.5 | 0.0 | 0.0 | 0.0 | 0.0 | 1.5 | 0.0 | 1.5 | 0.0 | 1.5 | 1.5 |

1. We include this combination in our analysis due the prevalence of the double certification Fairtrade/organic in the field. For each indicator, this combined standard shows the higher score of either the FLO or the EU Organic standard, reflecting the fact that under double certification farmers are bound by the rules of whichever standard is stricter. [↑](#footnote-ref-1)
2. The numbering “a” and “b” in this and all subsequent indicators indicate that they are mutually exclusive and/or that one indicator implicitly contains the other; they should thus not be summed when calculating maximum scores. [↑](#footnote-ref-2)
